# An in vivo wound healing model for the characterization of the angiogenic process and its modulation by pharmacological interventions

**DOI:** 10.1038/s41598-019-42479-1

**Published:** 2019-04-12

**Authors:** Martin Karl Schneider, Horea-Ioan Ioanas, Jael Xandry, Markus Rudin

**Affiliations:** Institute for Biomedical Engineering and Functional Pharmacology, ETH Zurich and University of Zurich, 8093 Zurich, Switzerland

## Abstract

Angiogenesis during wound healing is essential for tissue repair and also affected during cancer treatment by anti-angiogenic drugs. Here, we introduce a minimally invasive wound healing model in the mouse ear to assess angiogenesis with high spatiotemporal resolution in a longitudinal manner *in vivo* using two-photon microscopy in mice expressing GCaMP2 in arterial endothelial cells. The development of vascular sprouts occurred in a highly orchestrated manner within a time window of 8 days following wounding. Novel sprouts developed exclusively from the distal stump of the transsected arteries, growing towards the proximal arterial stump. This was in line with the incidence of Ca^2+^ transients in the arterial endothelial cells, most probably a result of VEGF stimulation, which were more numerous on the distal part. Functional analysis revealed perfusion across the wound site via arterial sprouts developed between days 6 and 8 following the incision. At day 8, proximal and distal arteries were structurally and functionally connected, though only 2/3 of all sprouts detected were actually perfused. Treatment with the FDA approved drug, sunitinib, the preclinical drug AZD4547, as well as with the combination of the two agents had significant effects on both structural and functional readouts of neo-angiogenesis. The simplicity and high reproducibility of the model makes it an attractive tool for elucidating migratory activity, phenotype and functionality of endothelial cells during angiogenesis and for evaluating specific anti-angiogenic drug interventions.

## Introduction

Proper function of cells and tissues in the body depend on sufficient supply by oxygen and nutrients via blood supply, i.e. on an efficient vascular system. While angiogenesis, the formation of new blood vessels, is highly active during development, it occurs in adulthood only in the cycling ovary and in the placenta during pregnancy or is reactivated during wound healing, though in general blood vessels are quiescent^1^. Under pathological conditions, however, may lead to activation of angiogenesis, e.g. in diseases such as wet macular degeneration, cancer, or inflammatory diseases^1,2^. During the past decades substantial research efforts have been undertaken to understand and modulate the angiogenesis process, which has led to the first FDA approved anti-angiogenic drug bevacizumab in 2004 against colorectal cancer. Bevacizumab is a monoclonal antibody against the vascular endothelial growth factor A (VEGF A), which has been identified as one of the essential growth factors driving angiogenesis^2^. Since then many other inhibitors of angiogenesis such as sunitinib, an inhibitor of the vascular endothelial growth factor receptor 1 and 2 (VEGFR-1, VEGFR-2), fetal liver tyrosine kinase receptor 3 (FLT3), KIT (stem-cell factor receptor), and platelet derived growth factor α and β (PDGFRα, and PDGFRβ) have been approved by the regulatory authorities for treatment of various cancers^3,4^. These new anti-angiogenic drugs have shown some beneficial impact on the survival outcome, but did not fulfil the high expectations so far. Limited efficacy and the development of resistance mechanisms remain critical obstacles to be resolved. New strategies involve the development of new drugs targeted against different growth factors and the combination of drugs to block escape mechanisms^1,5,6^. A promising drug candidate is AZD4547, a potent inhibitor of fibroblast growth factor-1, 2 and 3 receptor (FGFR-1, 2 and 3). Although AZD4547 has not been developed to specifically target angiogenesis signaling, FGF plays a crucial role in the angiogenesis process^7^.

Novel anti-angiogenic drug candidates are typically evaluated using animal models of human malignant cancer. Yet, given the high degree of temporo-spatial heterogeneity observed in tumor development, characterization of the angiogenic process and evaluation of its modulation by therapeutic interventions in a longitudinally manner and at microscopic resolution is intrinsically problematic. In fact, it has been shown that hypoxia signalling, one of the triggers of vessel outgrowth, and consequently the expression levels of its down-stream products, vary significantly across the tumor^8^. Hence, conditions, under which the angiogenic process is better confined both with regard to location and time would be preferable. This occurs, e.g. during wound healing, which involves the formation of neovessels to supply tissue distal to the wound. Following wounding the angiogenic signalling cascade is triggered within 2 to 3 days and deploys in an orchestrated/synchronized manner in spatial confined region close to the lesion^9^. Wound healing models are therefore attractive for studying the effect of pharmacological modulation of angiogenesis. The effect of anti-angiogenic therapy on wound healing is also clinically relevant as tumor therapy typically involves resection of the primary tumor. In many retrospective studies, a significant impact on the wound healing process has been shown in patients that received anti-angiogenic treatment, and guidelines have been established on how many days or weeks the respective anti-angiogenic treatment should be halted before and after the planed surgical procedure^10–12^.

Yet, monitoring the establishment of a vascular network during physiological wound healing in a mammalian model in a time-resolved manner at a microscopic resolution is challenging. All *in vivo* wound healing models in mouse are limited in resolving physiological angiogenic processes at microscopic resolution during the healing process, or require the implantation of a tissue window that constitutes a wound on its own and might lead to a non-physiological healing process due to insertion of the foreign material (window)^13–16^. In this study, we present a new *in vivo* model, which allows non-invasive monitoring of physiological wound healing at high temporal and spatial resolution in the mouse ear using a custom-made two-photon-microscope. We used the Cx40-GCaMP2 mouse, expressing the calcium indicator GCaMP2 in arterial endothelial cells (EC) under the control of the connexin40 (Cx40) promoter to study Ca^2+^ transients in activated endothelium^17^. Ca^2+^ transients are associated with activated VEGF receptors^18^. We analyzed morphological and functional changes during the healing process and studied its modulation using two anti-angiogenic drugs, sunitinib and AZD4547, both individually and as a combination therapy.

## Results

### Optimal time window for monitoring vessel formation

We chose to image the wound healing process from 3 days post transection (dpt) until 8 dpt. Literature data suggest onset of the angiogenesis process to occur at 3–4 dpt in wound healing^19^. Imaging before 3 dpt did not provide high-quality data due to the crust covering the wound. After 3 dpt the crust could be gently removed enabling high quality two-photon imaging. In the control group almost all transsected arteries were functional and perfused across the wound already at 6 dpt. Nevertheless, the observation period was extended to 8 dpt to ensure functional connections across the wound in all control mice and to quantitatively assess the improvement of perfusion over time. Extending the imaging period to 8 dpt was also relevant for investigating inhibitory effects of anti-angiogenic treatment on sprout formation and function. At time points beyond 8 dpt, the imaging quality also slightly decreased probably due to proliferation of fibroblasts and other cell types.

### Outgrow of arterial sprouts at distal but not proximal segments of transsected arteries

**Figure 1:**
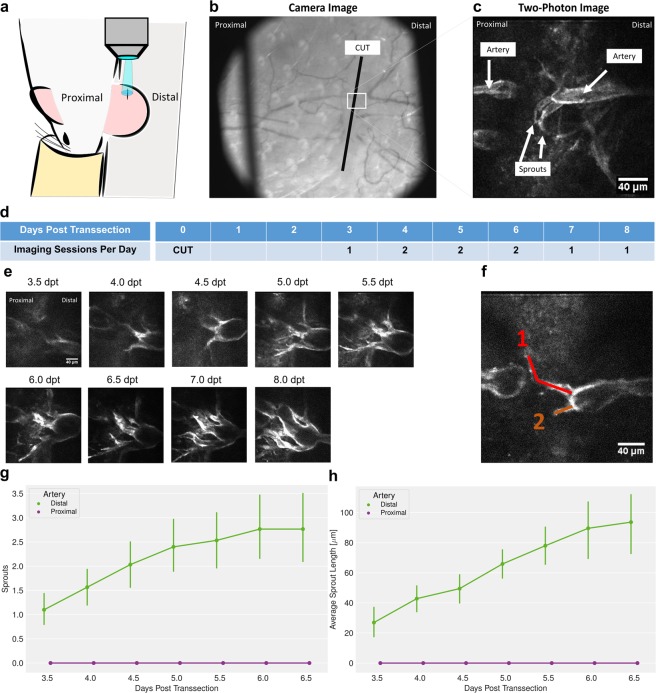
Angiogenic sprouting during wound healing. (**a**) Position of the ear during imaging and orientation of cut. (**b**) Overview picture of the cut, indicated by black line. (**c**) Representative two-photon image of the wound, showing sprouts growing from distal arteries stump towards the wound site. Only arterial vessel structures are visible due to selective expression of GCaMP2 in arterial endothelial cells. (**d**) Imaging sessions as a function time after incision (days post-transsection, dpt). (**e**) Representative two-photon images at as function of time following incision. (**f**) Evaluation of sprout length from transsected arteries. (**g**) Number of sprouts counted in control animals on the distal and proximal sides of the wound, from 3,5 to 6.5 dpt, n = 10. Error bars represent 90% confidence interval. No sprouts were detected on the distal side. The sprout count on the proximal side indicates a highly significant (p < 0.0005) mean increase of 1.5 +− 0.2 per dpt, based on the Laplace approximation to the posterior distribution of a Poisson generalized mixed model. (**h**) Average sprout length measured in control animals on the distal and proximal sides of the wound, from 3,5 to 6.5 dpt, n = 10. Error bars represent 90% confidence interval. No sprouts were detected on the distal side. The sprout lengths on the proximal side indicate a highly significant (p < 0.0005) mean increase of 10.4 +− 1.5 μm per dpt, based on a linear mixed effects model.

Arterial outgrow into the wound site of the mouse ear was monitored noninvasively using two-photon microscopy (dpt; Fig. 1a through e). First sprouts could be detected on the arterial segment distal to the wound at 3.5 dpt. For quantitative assessment of the angiogenic process, the number of sprouts was counted and the length of sprouts measured (Fig. 1f). All sprouts spreading towards the wound originated from the arterial stump distal to the wound (Fig. 1g,h); in none of the mice we observed any arterial sprouts at the proximal stump of the transsected arteries. There was a significant increase in number of sprouts from 3.5 dpt onwards to 6.5 dpt (Fig. 1g). Moreover, a steady significant increase of the sprout length has been found (Fig. 1h; Supplementary S1). After 6.5 dpt the majority of distal sprouts had reached the proximal site of the wound and formed functional perfused connections either directly to the proximal segment of the transsected arteries or to other vessels in the vicinity. While these vessels could not be directly visualized due to the limited depth penetration in two-photon microscopy, such connections could be inferred from blood flow measurements in the newly formed sprouts. Recordings at 7 or 8 dpt confirmed the establishment perfusion across the transsected arteries.

### Restoration of perfusion across wounds within 8 days

**Figure 2:**
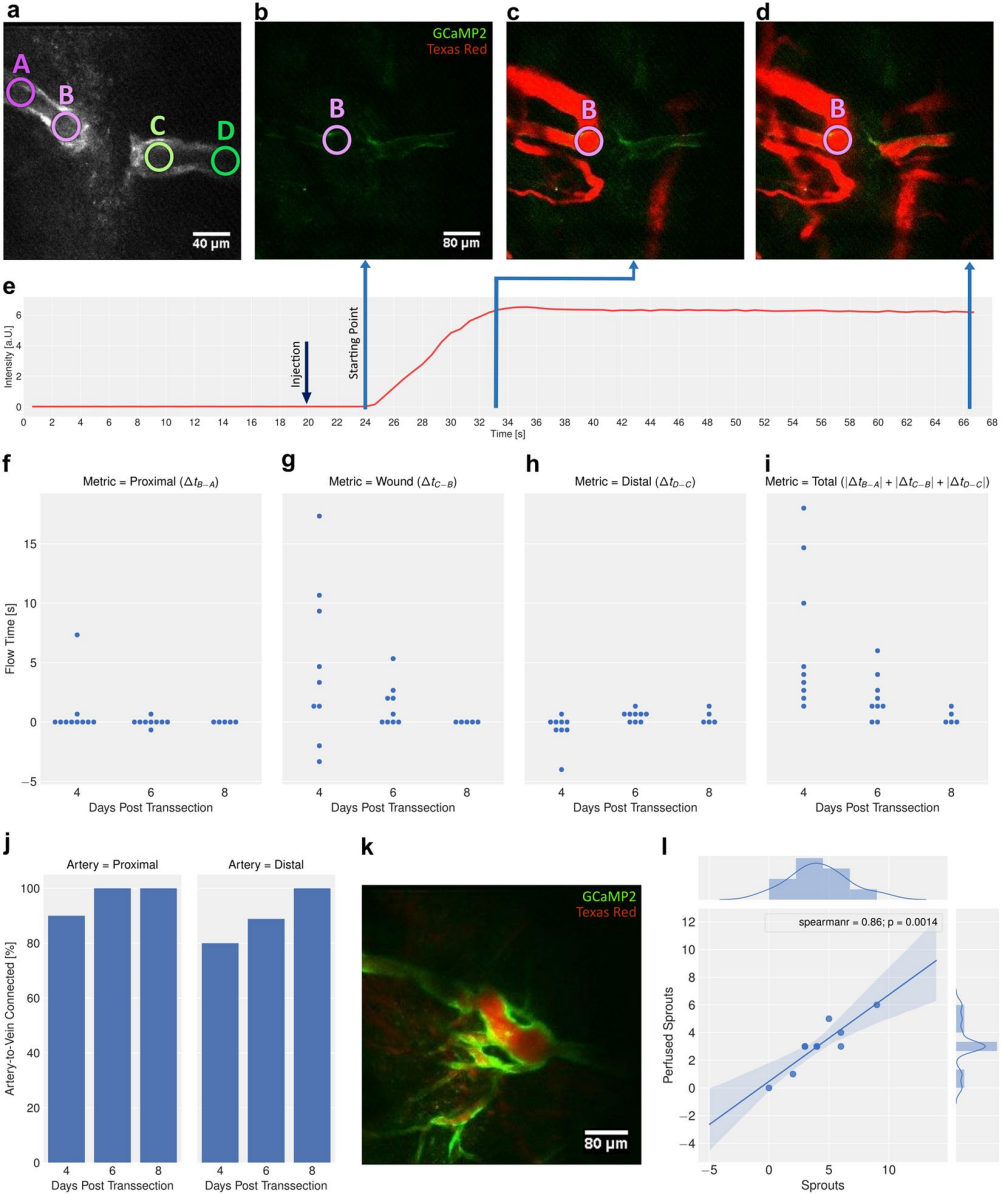
Recording of vessel perfusion as function of time for characterization of blood vessel functionality. (**a**) Overview image illustrating regions-of-interest (ROIs) inside the proximal stump (A,B) and the distal stump (C,D) of the transsected arteries. (**b**) Image recorded immediately prior to arrival of the injected red fluorescent tracer, Texas Red. (**c**,**d**) Images post-injection at time points indicated. (**e**) Measured intensity profile of red fluorescent signal during injection into the tail vein. (**f**) Quantification of proximal flow time ${\rm{\Delta }}{t}_{B-A}=t(B)-t(A)$ evolution over time (dpt). Statistical evaluation is based on a Mixed Linear Model Regression (MLMR) of treatment:time interactions. Significant time effects were observed in the control condition (p = 0.001, with a slope of −1.6 +− 0.5) and in the sunitinib treatment condition (p = 0.003, with a slope of 1.5 +− 0.5). (**g**) Quantification of wound flow time ${\rm{\Delta }}{t}_{C-B}=t(C)-t(B)$ as function of time (dpt). Statistical evaluation is based on MLMR of treatment:time interactions, yielded no significant time effect in the control condition (p = 0.316) with a slope of −1.1 +− 1.06. (**h**) Quantification of distal flow time ${\rm{\Delta }}{t}_{D-C}=t(D)-t(C)$ over time. Statistical evaluation is based on MLMR of treatment:time interactions, yielded significant time effect in the control condition (p = 0.028.) with a slope of 2.008 +− 0.916. (**i**) Integral flow measure $|{\rm{\Delta }}{t}_{B-A}|+\,|{\rm{\Delta }}{t}_{C-B}|+\,|{\rm{\Delta }}{t}_{D-C}$ as function of time (dpt). Statistical evaluation is based on MLMR of treatment:time interactions, yielded significant time effect in the control condition (p = 0.002) with a slope of −6.1 +− 1.9. (**j**) Analysis for anastomoses: Percentage of proximal and distal arteries connected to adjacent veins at 4, 6, 8 dpt (n = 10). (**k**) Combined GCaMP2/Texas Red imaging to assess perfused sprouts in compressed Z stacks. (**l**) Correlation between number of perfused sprouts and total number of sprouts. MLMR shows slope of 0.626 +− 0.061 (i.e. 62.6 +− 6.1% of sprouts are perfused, p < 0.0005). As values are discrete multiple data points overlap (total number of data points = 10).

The functionality of the newly formed sprouts/vessels was assessed using dynamic two-photon imaging during i.v. administration of the intravascular tracer Texas Red. The analysis revealed that under physiological/control conditions the proximal segment of the transsected arteries was well perfused in 90% of mice studied already at 4 dpt with a difference in tracer arrival time of ${\rm{\Delta }}{t}_{B-A}=t(B)-t(A) < 1.3s$. At 8 dpt all animals showed a ${\rm{\Delta }}{t}_{B-A}$ perfusion time <1 s (Fig. 2f). The analysis of the ROIs D-C placed into the distal segment of the transsected arteries revealed a difference in tracer arrival of ${\rm{\Delta }}{t}_{D-C} < \pm 1.3s$ in 80% of all mice studied (Fig. h). At 6 and 8 dpt none of the animals showed any differences in tracer arrival time exceeding 1.3 s (Fig. 2i). At 4 dpt we did not observe any blood flow across the wound, i.e. the tracer in the distal side of the wound arrived from distal end of the transsected arteries (${t}_{D-C} < 0$). At 6 dpt, average values ${\overline{{\rm{\Delta }}t}}_{D-C}\ge 0$ have been observed indicating the reestablishment of a functional connection between arterial trunks across the wound via newly formed sprouts (Fig. g; Supplementary S1).

Perfusion across the wound improved as a function of time with ${\rm{\Delta }}{t}_{C-B}$ decreasing from 4.74 ± 6.62 s at 4 dpt to 0 ± 0 s at 8 dpt (Fig. 2g). Accordingly, the sum $\,|{\rm{\Delta }}{t}_{B-A}|+\,|{\rm{\Delta }}{t}_{C-B}|+\,|{\rm{\Delta }}{t}_{D-C}|$, representing a measure for perfusion across the entire transected arteries, is decreasing until 8 dpt (Fig. 2i).

A striking observation was the efficient perfusion of proximal and distal segments of the transsected arteries already at 4 dpt with ${\rm{\Delta }}{t}_{B-A}\approx {\rm{\Delta }}{t}_{D-C} < 1.3s$ despite the lack of any connection between the two segments. We therefore analyzed for potential anastomoses between arterial trunks and adjacent veins, which could be differentiated as only arteries expressed GCaMP2. In fact, by analyzing the full z-stack we could connections from the arterial segment to a vessel not expressing GCaMP2, what we identified as connection from arteries to vein. Analyzing the proximal site of the wound revealed that 90% of the proximal arteries were connected to an adjacent vein (example Fig. 2d) at 4 dpt, and 100% at 6 and 8 dpt 100% in line with the small differences in tracer arrival measured. Similarly, for the distal site of the wound revealed that 80% and 100% of the distal transsected arteries were connected to adjacent veins on 4 dpt and 6 dpt (and beyond), respectively (Fig. 2j; Supplementary S1).

### More sprouts lead to more perfused blood vessels

Previous studies have shown that wound healing is accompanied by excessive sprouting of blood vessels, yet not every sprout was developing into a functional, perfused blood vessel^19^. Therefore, we compared the number of sprouts identified in structural images with the number of sprouts filled with Texas Red following the injection of the tracer (Fig. 2k). The correlation between the two measures revealed that approximately 60% of sprouts were perfused. The good correlation revealed a linear dependence within error limits, i.e. the ratio perfused/total number of sprouts remained constant throughout the observation period (Fig. 2l).

### Arterial structures distal to the wound site display higher Ca^2+^ activity than proximal ones

**Figure 3:**
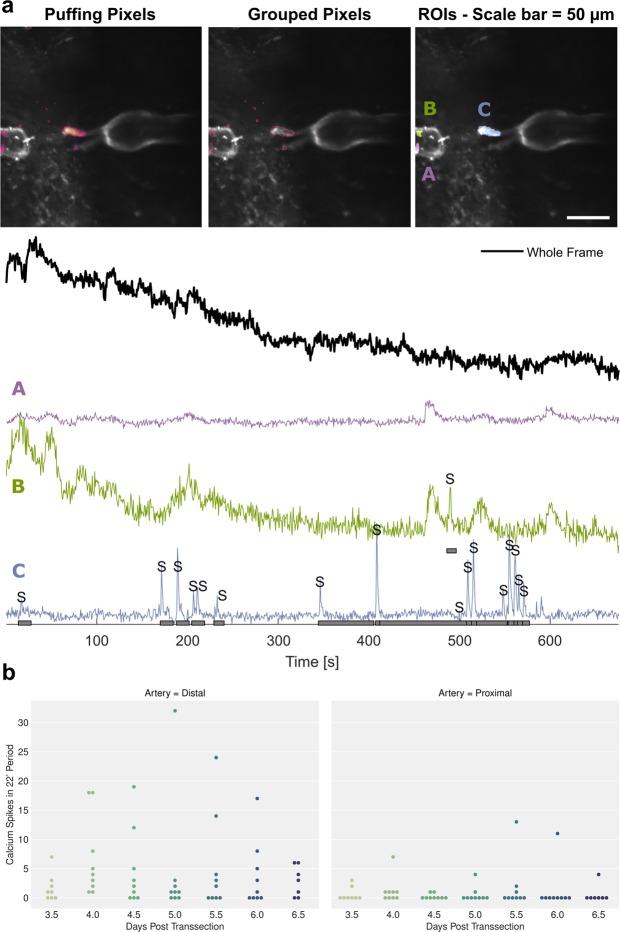
Analysis of calcium peaks under control condition for the period 3.5 dpt to 6.5 dpt. (**a**) Illustration of analysis procedure using software CHIPS. Top row: image frames showing grouped pixels as well as ROIs displaying identical calcium peak pattern. The four traces below indicate Ca^2+^ transient for a 11 min period spatially averaged across the entire picture (black) as well as Ca^2+^ transient for ROIs at the proximal arterial stump (A,B) and for sprouts emerging from the distal stump (C). Notice that clear Ca^2+^ transients were only identified for distal sprouts. (**b**) Number of Ca^2+^ transients during twice-daily 22 min recordings from 3.5 dpt to 6.5 dpt for proximal (left) and distal stump/sprouts (right) of transsected arteries. Wilcoxon signed-rank test revealed signal difference between number of distal versus proximal Ca^2+^ transients (p = 0.00001).

The growth of blood vessels is associated with expression and secretion of VEGF from hypoxic tissue like wound tissue. It has been shown that VEGF through binding to its VEGF-receptor is triggering a calcium influx into endothelial cells^18^. We therefore assessed the occurrence of arterial endothelial Ca^2+^ transients in the vascular segments around the wound over a period of 22 min at each imaging time point (3.5 to 6.5 dpt) during the wound healing process. Data analysis using the software program CHIPS (Fig. 3a)^20^ yielded the number of Ca^2+^ transients that occurred during the observation period as well as their anatomical location. The results showed the Ca^2+^ transients predominantly occurred in arterial structures distal to the wound, in particular in the sprouts. Statistical analysis using the Wilcoxon signed rank test revealed a significant difference in the number of Ca^2+^ transients detected: the number of transients on proximal transsected arteries was significantly lower than that on the distal transsected arteries (n = 9, p < 0.00001). The higher incidence of calcium peaks on the distal transsected arteries correlates with its significantly higher sprouting activity (Fig. 1). The time dependence of the Ca^2+^ transients indicates an increase in frequency between days 3 and 5 followed by a decrease between days 5 and 8.

### Treatment with anti-angiogenic drugs reduces number of sprouts, sprout length and impairs perfusion to and across the wound

**Figure 4:**
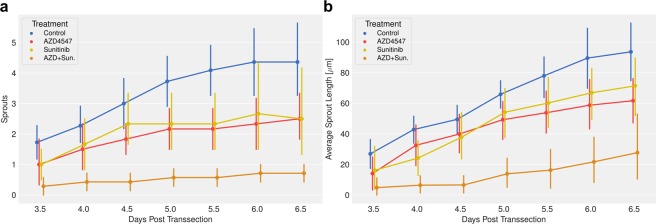
Monotherapy and combined treatment with anti-angiogenic drugs reduces number and average lengths of sprouts. Mice treated with either AZD4547 (40 mg/kg/day) or sunitinib (40 mg/kg/day), or with the combination with AZD4547 + sunitinib (40 mg/kg/day each). Treatment was initiated at 1 dpt and continued until the end of the experiment. (**a**) Number of distal sprouts as function of time for period 3.5 to 6.5 dpt. (**b**) Average sprout length as function of time for period 3.5 to 6.5 pdt. Statistical evaluation is based on Mixed Linear Model Regression of the treatment:time interaction, with omnibus ANOVA F(3,436) = 8.55 and p = 0.00016, and Holm-Bonferroni corrected post-hoc t-tests ****P ≤ 0.0001. Data are center points represent the mean, and the error bars represent 90% confidence intervals. (n = 10 control; n = 6 sunitinib; n = 6 AZD4547; n = 7 AZD4547 + sunitinib). Color code: Treatment groups Control = BLUE; AZD4547 = RED; Sunitinib = GOLD; AZD + Sun = ORANGE.

To assess the effect of anti-angiogenic therapy on wound healing, mice were treated with sunitinib, AZD4547, as well as the combination AZD4547 + sunitinib from 1 dpt until 8 dpt (termination of experiment). Similar to the findings in control mice, sprouting was only observed from the distal segment of the transsected arteries, irrespective of the treatment (Fig. 4a,b). Treatment with either sunitinib or AZD4547 alone led to a reduction of the number of sprouts, the difference to control group becoming significant as of 5.5 dpt. This effect was more pronounced in mice receiving the combination therapy, AZD4547 + sunitinib. Under this combined treatment the number of sprouts were already significantly suppressed from 4.0 dpt onwards. However, the differences between the three treatment groups did not reach statistical significance (Fig. 4a).

Similar results have been found for the average sprout lengths: Treatment with sunitinib or AZD4547 alone led to a significant reduction of the average sprout length compared to the control mice, which became significant as of 6.0 dpt. Again these effects were more pronounced by the combination treatment AZD4547 + sunitinib, for which significant differences to the control group were already observed as of 4.0 dpt, and to the monotherapy groups as of 5.0 dpt (Fig. 4b).

**Figure 5:**
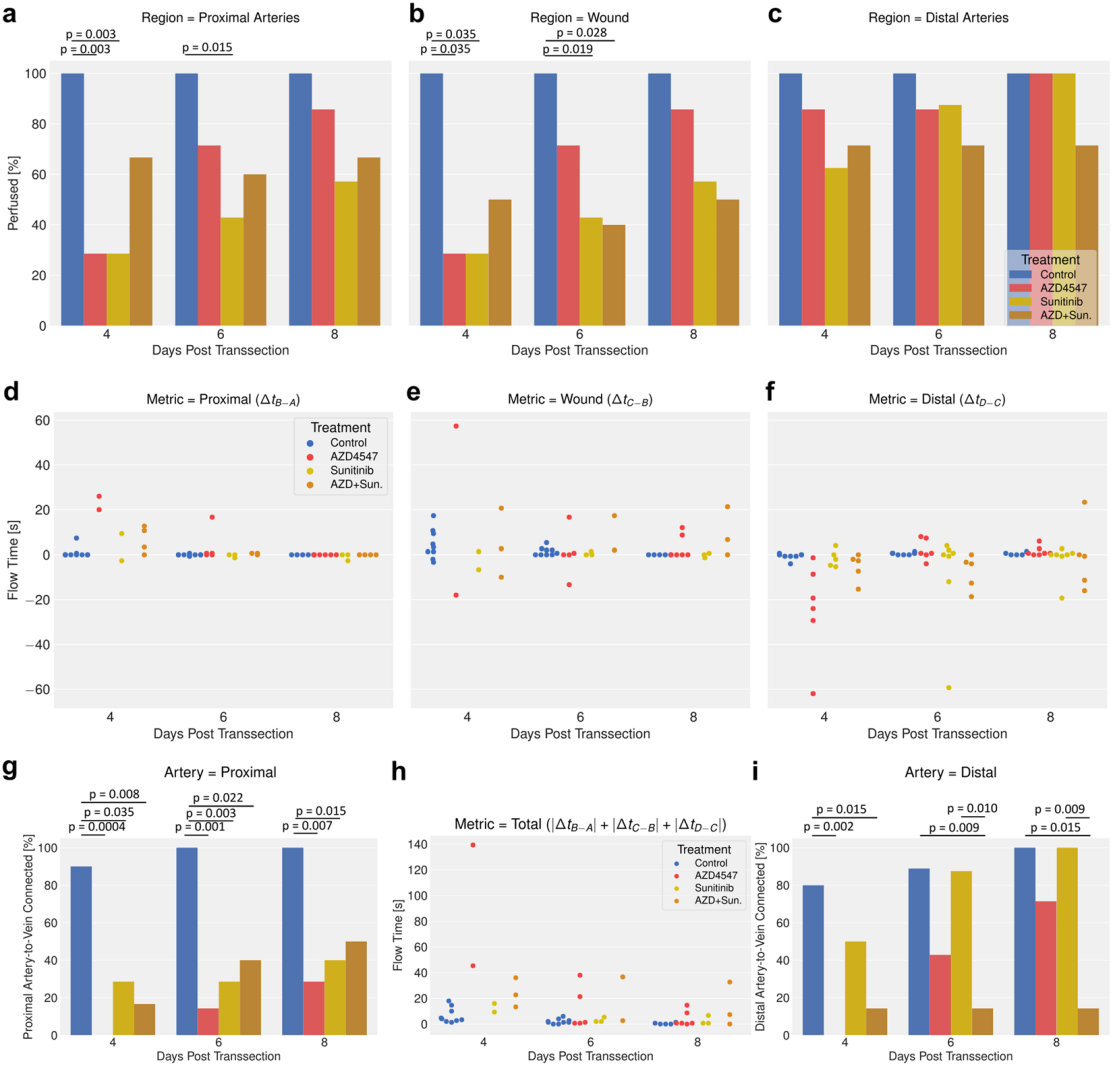
Monotherapy or combined drug treatment with anti-angiogenic drugs affects perfusion of proximal and distal transsected arteries at 4, 6 and 8 dpt. (**a**–**c**) Percentage arteries displaying perfusion (uptake of Texas Red) as function of time. (**a**) Proximal arteries ${\rm{\Delta }}{t}_{B-A} < 66.66s$. (**b**) Across the wound perfused ${\rm{\Delta }}{t}_{C-B} < 66.66s$. (**c**) Distal perfused arteries ${\rm{\Delta }}{t}_{D-C} < 66.66s$; Statistical comparison: Fisher’s exact test; *P < 0.05; **P < 0.01. (**d**,**f**) Mean tracer transit time. Error bars represent 95% confidence interval. Statistical evaluation is based on Mixed Linear Model Regression of the treatment:time interaction. (**d**) Proximal arterial stump ${\rm{\Delta }}{t}_{B-A}$. Omnibus ANOVA F(3,47) = 1.54 and p = 0.217(all Holm-Bonferroni corrected post-hoc t-tests p > 0.05). (**e**) Across wound ${\rm{\Delta }}{t}_{C-B}$. Omnibus ANOVA F(3,47) = 1.02 and p = 0.39 (Holm-Bonferroni corrected post-hoc t-tests p > 0.05). (**f**) Distal arterial stump ${\rm{\Delta }}{t}_{D-C}$. Omnibus ANOVA F(3,47) = 1.32 and p = 0.28 (Holm-Bonferroni corrected post-hoc t-tests p > 0.05). (**h**) Total perfusion index $|{\rm{\Delta }}{t}_{B-A}|+\,|{\rm{\Delta }}{t}_{C-B}|+\,|{\rm{\Delta }}{t}_{D-C}$ as function of time. Omnibus ANOVA F(3,47) = 1.65 and p = 0.19 (Holm-Bonferroni corrected post-hoc t-tests p > 0.05). (**g**,**i**) Percentage of arteries connected to their adjacent veins (anastomoses). (**g**) Proximal anastomoses. (**i**) Distal anastomoses. Statistical comparison: Fisher’s exact test; *P < 0.05; **P < 0.01. Color code: Treatment groups Control = BLUE; AZD4547 = RED; Sunitinib = GOLD; AZD + Sun. = ORANGE.

The drug induced reduction in sprout number and average sprout length led to impaired perfusion values across and around the wound site. In a significant fraction of drug treated mice the tracer did not reach the proximal segment of the transsected arteries during the measurement interval of 46.7 s, although the tracer reaches other vessels at the proximal side of the wound. In fact, 2/7 mice of either the sunitinib or AZD4547 group showed perfused proximal arteries at 4 dpt, significantly less than in the control group. These numbers increased for both groups as a function of time with 3/6 and 6/7 mice showing perfusion at 8 dpt, respectively. Interestingly, for the combination treatment, 4/6 mice did show tracer uptake in the proximal arterial stump at 4 dpt, though this value did not change over time. Over the whole imaging period of 8 dpt none of the treated mice groups reached 100% perfused proximal arteries, in contrast to the control group for which showed already 90% perfused proximal arteries at 4 dpt and full perfusion at 6 and 8 dpt (Fig. 5a).

We observed a slight reduction in the number of perfused distal arteries in all treated groups in comparison to the control mice over the whole imaging period of 8 dpt. The number of distal perfused arteries in the AZD4547 or sunitinib group was slightly, but not significantly reduced, and recovered until 8 dpt completely. Such a recovery was not observed in the group receiving combination treatment (Fig. 5c). The ROIs C-B next to the wound site yielded essentially the same information like proximal ROIs B-A. Only the combined treatment showed an significant reduction at 6 dpt (Fig. 5b).

For mice showing perfusion of transsected arterial stumps blood flow based on tracer arrival times was analyzed quantitatively, though given the large fraction of animals displaying no perfusion at all statistical analysis was compromised by small numbers. In mice receiving mono– or combination therapy, there was a trend towards reduced blood flow in the proximal arterial stump at 4 dpt (i.e. ${\rm{\Delta }}{t}_{B-A}({\rm{drug}}) > {\rm{\Delta }}{t}_{B-A}({\rm{control}}))$, with perfusion largely normalized at 8 dpt (Fig. 5d). Similar results have been obtained for the distal arterial stump: compromised perfusion at 4 dpt, with some trend towards recovery for the monotherapy groups. Perfusion values in mice receiving combination therapy remained low throughout the observation period. Negative flow times, indicating flow direction from distal to proximal, were found predominantly in the distal arterial segment (${\rm{\Delta }}{t}_{D-C} < 0)$ and across the wound ${\rm{\Delta }}{t}_{C-B} < 0)$ (Fig. 5e,f).

A critical step in the wound healing process is re-establishment of perfusion across the wound, i.e. the arterial sprouts connecting the proximal and distal stump have to be functional. We therefore analyzed the effect of drug administration on the difference of tracer arrival time in the ROIs B and C, i.e. ${\rm{\Delta }}{t}_{C-B}$. The mice receiving mono– or combination therapy, tended to show a compromised blood flow at 4 dpt (i.e. ${\rm{\Delta }}{t}_{C-B}({\rm{drug}}) > {\rm{\Delta }}{t}_{C-B}({\rm{control}}))$, an effect that was largely normalized at 8 dpt for the mono-therapy groups, while it persisted for the mice receiving the combination therapy throughout the observation period (Fig. 5e). The combined perfusion readout defined as the sum of the individual measures on the arterial segments and the wound itself, i.e. ${\rm{\Delta }}t=|{\rm{\Delta }}{t}_{B-A}|+\,|{\rm{\Delta }}{t}_{C-B}|+\,|{\rm{\Delta }}{t}_{D-C}|$ revealed similar effects of drug treatment as described for ${\rm{\Delta }}{t}_{C-B}$ (Fig. 5h).

High-resolution intravital imaging showed that treatment with AZD4547 or sunitinib alone suppressed the formation of functional anastomoses between the proximal arterial stump and an adjacent vein significantly. Some anastomoses developed between 4 and 8 dpt, though this effect was not significant. Similarly, combined AZD4547 + sunitinib treatment resulted in significantly less functional anastomoses on 4 and 6 dpt in comparison to the control (Fig. 5g).

For the distal arterial stump findings were essentially identical. Monotherapy led to a reduced number of anastomoses, though the effect reached only significance for mice receiving AZD4547 at 4 dpt when compared to controls. Subsequently, anastomoses between the distal arterial stump and adjacent veins developed very well, in particular for the sunitinib group (100% at 8 dpt). Also the AZD4547 group showed a fast recovery until 8 dpt. For mice receiving combination therapy, a significantly reduced number of distal anastomoses was observed throughout the whole observation period (Fig. 5i; Supplementary S1-S4).

### Drug treatment did not affect the fraction of perfused sprouts

**Figure 6:**
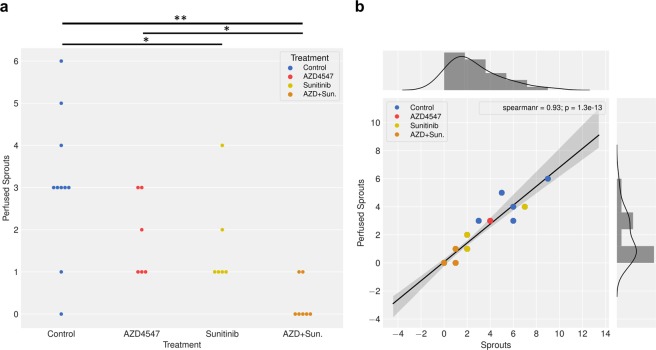
Anti-angiogenic therapy does not affect ratio of perfused versus total number of sprouts. (**a**) Number of perfused sprouts at 8 dpt. (n = 10 control; n = 8 sunitinib; n = 7 AZD4547; n = 7 AZD + Sun) Statistical comparison: Mann-Whitney U test. *p < 0.05; **p < 0.01. (**b**) Correlation between number of perfused sprouts and total number of sprouts. MLMR shows slope of 0.673 +− 0.036 (i.e. 67.3 +− 3.6% of sprouts are perfused). As values are discrete, multiple data points overlap (total number of data points = 29). Color code: Treatment groups Control = BLUE; AZD4547 = RED; Sunitinib = GOLD; AZD + Sun. = ORANGE.

A critical readout for assessing the effects of antiangiogenic drugs on wound healing is the functionality of the newly formed sprouts. As already discussed, both mono and combination treatment reduced the number of sprouts. Concomitantly, we observed significantly less perfused sprouts at day 8 in all groups. The effect was even more pronounced in mice receiving combination treatment (Fig. 6a). Correlation analysis revealed that anti-angiogenic therapy did not affect the ratio of perfused sprouts/total sprouts within error limits, i.e. approximately 2/3 of the newly formed vessels were functional (Fig. 6b; Supplementary S1-S4).

## Discussion

We have introduced a minimally invasive wound healing model in the mouse ear to assess angiogenesis with high spatiotemporal resolution in a longitudinal manner *in vivo* using two-photon microscopy. The use of transgenic mice expressing fluorescent GCaMP2 in arterial endothelial cells allowed studying neo-vessel formation originating exclusively from arterial structures. We observed that the development of vascular sprouts occurred in a highly orchestrated manner within a time window of 8 days following cut. This time course correlates well with published data^19,21^. The high spatiotemporal resolution achieved by intra-vital microscopy provided detailed insights into the process of vessel formation. Novel sprouts developed exclusively from the distal stump of the transsected arteries, growing towards the proximal arterial stump. At this stage we can only speculate about the reasons of this asymmetry. A likely explanation might be that the area distal to the wound becomes transiently hypoxic triggering HIF signaling and the secretion of VEGF, though additional experiments will be required to clarify this point. The asymmetry between proximal and distal sprouting was also revealed by the analysis of the Ca^2+^ transients, which reflect activation of arterial endothelial cells probably in response to stimulation by VEGF, as Ca^2+^ transients have been associated with activated VEGF receptors^18^. Ca^2+^ transients thus provide an early indication of angiogenic signaling. Interestingly, the frequency of Ca^2+^ transients increased from day 3 on to reach a maximum around day 5 to decrease again to baseline values around day 8 following cut, which mimics the dynamics of sprout development. The frequency of Ca^2+^ spikes was low in average. Around day 5 following the cut we measured in average 5 transients for a 22 min period, i.e. 1 spike every 4 to 5 min, which is significantly lower compared to what has been reported for Zebra fish embryos^18^. Apparently, blood endothelial cells are rather quiescent in adult animals^1^. The low incidence of Ca^2+^ spiking in adult GCaMP2 mice and thus the small dynamic range for assessing modulatory effects renders it insensitive for evaluating drug effects on vessel neoformation.

A critical aspect when studying angiogenesis is the functionality of the newly formed blood vessels, which can be studied by analyzing the arrival of an intravascular fluorescent tracer (Texas Red) using dynamic two-photon imaging. Several mechanisms may contribute to reperfusion of the transsected arteries: The formation of anastomoses to nearby veins at both the proximal and distal side of the wound, retrograde perfusion of the distal arterial stump as well as flow through arterial sprouts that have connected to proximal vessels across the wound. Analysis of tracer arrival times in the various vascular segments indicated contributions from all three mechanisms. Already on 4 dpt very fast tracer passage with a transit time of <1.3 s could be measured across the proximal and distal wounded arterial segments. As at this time there were no functional sprouts connecting the arterial stumps, the observation clearly hints at newly established anastomoses to adjacent veins on the respective side of the wound. This interpretation is supported by direct observation of these arterial-venous shunts. On the distal side of the wound, we typically observed retrograde perfusion in the distal arterial stump, i.e. blood flowing from the distal towards the proximal end of the vessel indicating reverted perfusion pressure. Perfusion across the wound site via arterial sprouts developed between days 5 and 8 following the incision. At day 8 proximal and distal arteries were structurally and functionally connected, though only 2/3 of all sprouts detected were actually perfused.

Treatment with the FDA approved drug sunitinib^4^, the preclinical drug AZD4547^7^, as well as with the combination of the two agents had significant effects on both structural and functional readouts of neo-angiogenesis. Both mono-therapies significantly reduced the number and average length of sprouts, yet at the doses used, did not prevent successful reperfusion across the wound at day 8 following the incision. The asymmetry in the angiogenic sprouting behavior between proximal and distal side of the wound is preserved in the drug treated animals. These data show that anti-angiogenic therapy via VEGF and FGF inhibition prolonged the wound healing process, but did not prevent it. This effect was even more pronounced for the combination therapy AZD4547 + sunitinib, which largely prevented the development of functional sprouts during the observation period, though there was a trend towards normalization. This finding is in line with clinical observations obtained from retrospective studies of patient treated with anti-angiogenic drugs, such bevacizumab. VEGF inhibition led to a significant increase in the incidence of wound healing complications but by far not every patient was affected^10^.

Interestingly, the effect of drug treatment on the formation of anastomoses is more pronounced on the proximal than on the distal side of the wound. This might indicate a gradient in drug concentration from proximal to distal and/or a gradient in angiogenic signaling, i.e. in the concentration of proangiogenic factors such as VEGF and FGF. The fact that both Ca^2+^ signaling and sprouting activity are significantly higher at the distal side of the wound hint in this direction. Higher drug levels at lower baseline concentration of pro-angiogenic factors might explain the stronger drug effect on proximal anastomoses. Interestingly, the combination treatment AZD4547 + sunitinib largely prevented the formation of distal anastomoses as well, likely due to the higher drug concentration and the fact that several pro-angiogenic factors are simultaneously inhibited. Drug treatment did not affect the fraction of perfused sprouts, i.e. the ratio # perfused sprouts/# total sprouts indicating that anti-angiogenic treatment reduced the number of sprouts but not their intrinsic nature.

In conclusion, we have characterized a novel wound healing model for assessing neo-angiogenesis non-invasively at high temporospatial resolution using two-photon microscopy. The wound consists of a small cut in the ear of a mouse, in our case mice that expressed the Ca^2+^ indicator GCaMP2 in arterial endothelial cells, which allowed monitoring Ca^2+^ transients as indicator of activated endothelium. Following the incision, angiogenesis was found to occur in a highly orchestrated manner over a time period of 3–8 days. Arterial sprouts were found to develop exclusively form at the distal stump of a transected arteries, growing towards the proximal arterial stump. This is in line with observation of Ca^2+^ transient in arterial endothelial cells, which were predominantly observed at the distal side of the wound. At 8 days following the incision, functional connection between proximal and distal arterial stumps via the sprouts was fully established. Approximately 2/3 of the sprouts were found to be functional. Anti-angiogenic therapy using VEGF and FGF inhibition was found to reduce the average number and length of sprouts, delaying but not preventing functional connection across the wound. Combining the two drugs showed a larger inhibitory effect. The simplicity and high reproducibility of the model makes it an attractive tool for elucidating migratory activity, phenotype and functionality of ECs during angiogenesis and for evaluating specific anti-angiogenic drug interventions.

## Material and Methods

### Two-Photon Microscope

Images were acquired using a custom-built two-photon laser-scanning microscope^22^. A 20x water immersion microscope objective was used (W Plan-Apochromat 20x/1.0 DIC VIS-IR, Carl Zeiss AG, Feldbach, Schweiz). GCaMP2 and Texas Red were both excited at 900 nm with an InSight DeepSee laser (Newport Spectra Physics, Darmstadt, Germany) with a laser power between 10 and 30 mW. Fluorescence emission was detected with a GaAsP photomultiplier module (Hamamatsu Photonics Deutschland GmbH, Herrsching, Germany) with a band-pass filter BrightLine HC 535/50 and a BrightLine 630/69 (LLC Semrock Inc., Rochester, USA). The two-photon laser-scanning microscope was controlled by a customized version of “ScanImage” (r3.8.1; Janelia Farm Research Campus, Ashburn VA, USA).

### Animals

Cx40-GCaMP2 mice^17^ were obtained from M.Kotlikoff, Cornell University and the CHROMus mouse resource (R24HL120847) and were housed under pathogen-free conditions until imaging. To achieve a better light penetration depth with the 2-photon microscope, Cx40-GCaMP2 mice, which are bread on a C57Bl/6 background, were crossed with Bl6 albino mice. Genotyping of the Cx40-GCaMP2-Bl6/albino was carried out using PCR (RP24-3229-18bp 5′ AAT ACA ACG GCT ATC ACG; RP24-19bp-3823 5′ AGC TTC TGG CTT TCT TTA C). Female Cx40-GCaMP2-Bl6/albino mice at the age of 7–25 weeks were used for *in vivo* imaging studies. All experiments were carried out in strict adherence to the Swiss Law of Animal Protection according to the ARRIVE guidelines and approved by the Kantonales Veterinaeramt Zurich (protocols: 185/2013 and 161/2016).

### Drug treatment

40 mg/kg/day AZD4547 and 40 mg/kg/day sunitinib (both selleckchem, Boston, USA) were dissolved in 10% DMSO + 30% Polyethylene glycol (both Sigma-Aldrich, Darmstadt, Germany) and dH_2_O. The solution was given per oral gavage.

### Intravital imaging

For surgery, mice were anesthetized by exposing them to 3% isoflurane in an induction chamber, and anesthesia maintained by continuous inhalation 2% isoflurane in 70%air/30%oxygen delivered via a face mask. To maintain the body temperature at 37 ± 0.5 °C, mice were placed on a heating pad. The ear was fixed on a custom-made aluminum support. A scalpel was used to cut a small wound on the dorsal side of the ear of about 3 ± 0.5 mm in length and 0.3 ± 0.1 mm in depth. Care was taken not to injure the cartilage layer (Fig. 1). Three days after the cut, first imaging experiments were carried out. For imaging, mice were again anesthetized with 3% isoflurane in an induction chamber, and anesthesia was maintained by inhalation of 1, 5–2% isoflurane delivered via a face mask. The ear was positioned on custom-made aluminum support and fixed with a 25 × 50 mm coverslip, which was attached to the aluminum support with a double-sided adhesive tape. Care was taken to minimize pressure on the ear to minimize effects on local circulation. The gap between the coverslip and the ear was filled up with deionized water, which was also used as coupling medium between the objective and coverslip. Three sets of images were recorded: (1) Anatomical images by recording 3D imaging cubes (stacks), (2) Dynamic 2D imaging for a slice selected from the 3D stack to record Ca^2+^ transients, (3) Perfusion imaging by dynamically recording of the passage of a intravascular fluorescent tracer.

#### 3D anatomical imaging

For 3D anatomical imaging the in-plane field-of-view (FOV) was 1 × 1 mm^2^ and the digital resolution was set to 256 × 256 or 128 × 128 pixels, respectively. 32 slices were recorded in the z-direction (depth) with an interslice distance of 2 μm. Acquisition of a full 3D stack took in average 32 s. Arteries and arterioles were identified on the basis of the intrinsic weak GCaMP2 activity.

#### Dynamic recording of Ca^2+^ transients

Based on the 3D data stack, a slice of interest showing arterial sprouts was selected. Imaging parameter for the 2D slice were FOV = 1 × 1 mm^2^, digital resolution = 256 × 256 pixel and acquisition time = 0.66 s/image. Image series with 2000 sequential images covering a period of 22 min were recorded.

#### Dynamic imaging of vessel perfusion studies

Perfusion studies were carried out at 4, 6, and 8 days following transsection. A stack of 2D slices was recorded using a FOV = 1 × 1 mm^2^, digital resolution = 128 × 128 pixels, and 40 sections in z-direction with an interslice distance of 2 μm. 100 sequential data sets were recorded at a frame rate of 0.66 frames/s. In case the transsected arteries were not perfused within the first 100 frames, a total of 300 sequential frames were recorded. After recording 30 frames as a baseline, 200 ug Texas Red Dextran (Thermo Fisher Scientific AG, Reinach, Switzerland) dissolved in 100 ul NaCl was injected into the tail vein.

### Image processing

Sprouts number and sprout length was analyzed manually with Fiji (NIH, LOCI, Wisconsin, USA). Sprout length was determined by measuring the distance between the wall of the wounded arteries and the tip of the sprouts within the whole Z-stack over 6.5 days wound healing time course.

Calcium transients were detected and analyzed by custom-designed image processing tool box for MATLAB (Cellular and Hemo- dynamic Image Processing Suite (CHIPS; Barrett *et al*., 2018; R2014b^20^; MathWorks).

Perfusion analysis: The minimum recording time was 66,7 s (100 frames). Prior to tracer injection, baseline images were recorded for 20 seconds (0.66 frames/s) (Fig. 2b through e). Following tracer administration, images were recorded for at least 66,7 s (100frames). For quantitative analysis of vessel perfusion four different region of interest (ROI; A-B-C-D) were placed from proximal to distal inside the lumen of the transsected arteries. ROIs A and B were placed within the proximal transsected arteries and ROIs C and D inside the distal arteries (Fig. 2a). The time point of tracer arrival (intensity change) in ROI *i* (*i* = *A*,*BC*,*D*) was defined as time *t*(*i*). The time difference was calculated as ${\rm{\Delta }}{t}_{j-i}=t(j)-t(i)$ with pairs (*i*,*j*) = (*A*,*B*),(*B*,*C*),(*C*,*D*), respectively.

The presence of functional anastomoses was defined on the basis of manual Z-stack analysis. Time delays of ${\rm{\Delta }}{t}_{B-A}$ or ${\rm{\Delta }}{t}_{D-C}$ < 1.3 s indicated an anastomosis.

### Statistical analysis

Plots were generated using the free and open source matplotlib^23^ and Seaborn^24^ libraries. Modelling and statistical analysis including Mixed General Linear Models (GLM), Analysis of Variance (ANOVA), t-tests, Holm-Bonferroni corrections, Wilcoxon signed-rank tests, and Pearson’s and Spearman’s r calculation were performed with the free an open source libraries Statsmodels^25^ and SciPy^26^. All code used to generate plots and top-level statistics (along with the top-level data) has been made openly accessible^27^.

Fisher’s exact test was performed with GraphPad Prism 7 (GraphPad Prism Software). N, number of independent experiments. n.s., non-significant; *p < 0.05; **p < 0.01; ***p < 0.001; ****p < 0.0001.

## Supplementary information

An in vivo wound healing model for the characterization of the angiogenic process and its modulation by pharmacological interventions

S1 - S4
