# Supplementary material for: An in vivo wound healing model for the characterization of the angiogenic process and its modulation by pharmacological interventions
Source: Sci Rep. 2019 Apr 12;9:6004. doi: 10.1038/s41598-019-42479-1 (PMC6461656; doi:10.1038/s41598-019-42479-1)
Supplement: Supplementary file 1 — An in vivo wound healing model for the characterization of the angiogenic process and its modulation by pharmacological interventions [file 41598_2019_42479_MOESM1_ESM.docx]

**An in vivo wound healing model for the characterization of the angiogenic process and its modulation by pharmacological interventions**

**Martin Karl Schneider^1^, Horea-Ioan Ioanas^1^, Jael Xandry^1^, Markus Rudin^1*^**

***Correspondence:**

**Prof. Dr. Markus Rudin**

**Institute for Molecular Imaging and Functional Pharmacology**

**University and ETH Zurich**

**HIT E22.4**

**Wolfgang-Pauli-Strasse 27**

**CH-8093 Zurich**

**Switzerland**

**Rudin@biomed.ee.ethz.ch**

**1:** Institute for Biomedical Engineering and Functional Pharmacology, ETH Zurich and University of Zurich, 8093 Zurich Switzerland

**Supplementary Information**

**Figure Legends Supplemental Figures**

**Figure S1**. Related to Figure 2. Angiogenic sprouting under control conditions. (a) Representative two-photon images at as function of time following incision (b) Recording of vessel perfusion at 4,6 and 8 dpt. (c) Z-stack after tracer, Texas Red, injection.

**Figure S2**. Related to Figure 2. Angiogenic sprouting under AZD4547 treatment. (a) Representative two-photon images at as function of time following incision (b) Recording of vessel perfusion at 4,6 and 8 dpt. (c) Z-stack after tracer, Texas Red, injection.

**Figure S3**. Related to Figure 2. Angiogenic sprouting under sunitinib treatment. (a) Representative two-photon images at as function of time following incision (b) Recording of vessel perfusion at 4,6 and 8 dpt. (c) Z-stack after tracer, Texas Red, injection.

**Figure S4**. Related to Figure 2. Angiogenic sprouting under AZD4547+sunitinib treatment. (a) Representative two-photon images at as function of time following incision (b) Recording of vessel perfusion at 4,6 and 8 dpt. (c) Z-stack after tracer, Texas Red, injection.
