# Supplementary material for: An in vivo wound healing model for the characterization of the angiogenic process and its modulation by pharmacological interventions
Source: Sci Rep. 2019 Apr 12;9:6004. doi: 10.1038/s41598-019-42479-1 (PMC6461656; doi:10.1038/s41598-019-42479-1)
Supplement: Supplementary file 2 — S1 - S4 [file 41598_2019_42479_MOESM2_ESM.zip › supplementary_Videos_included/S4_AZD4547+Sunitinib.pptx]

## Slide 1
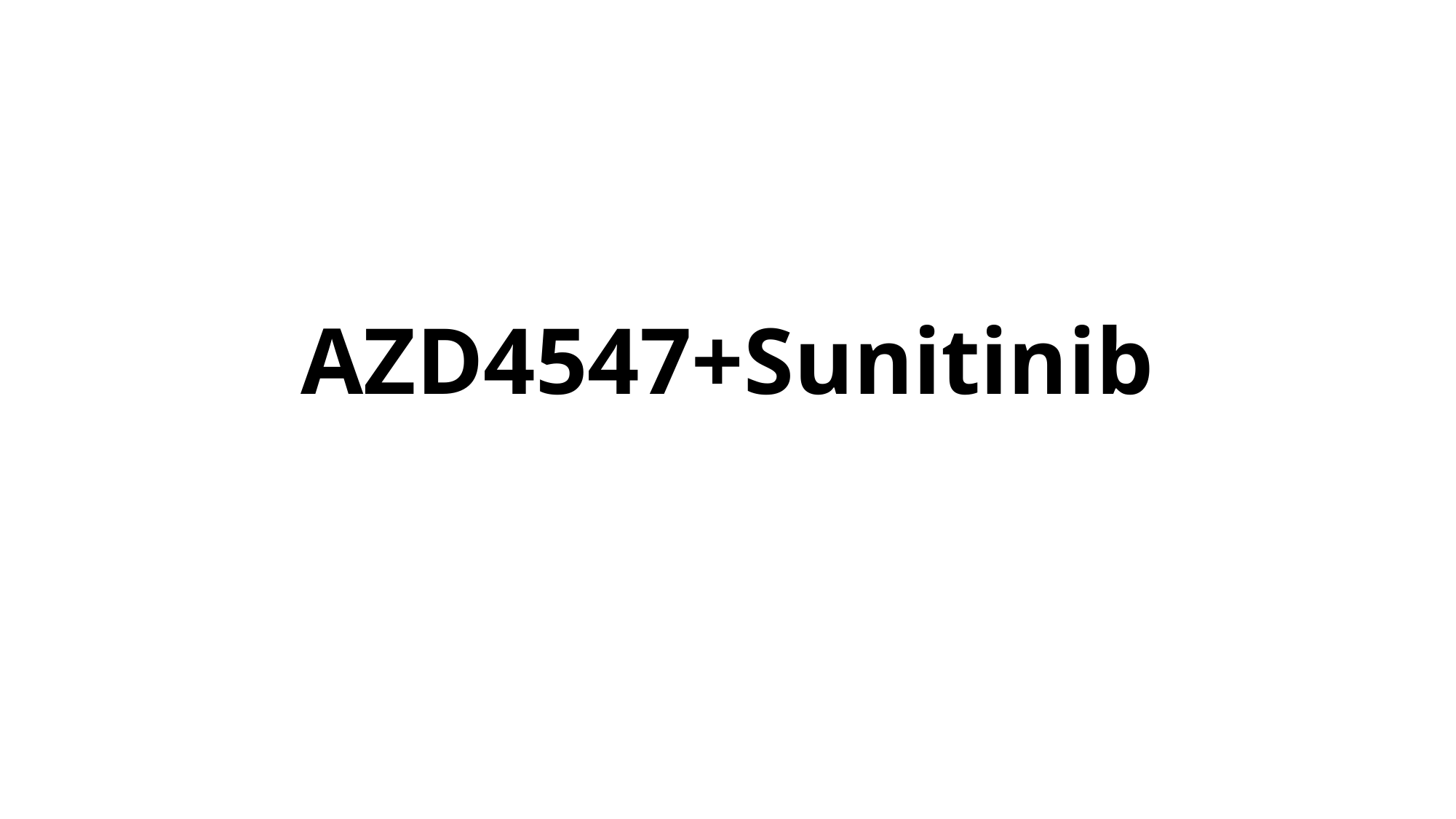

# AZD4547+Sunitinib

## Slide 2
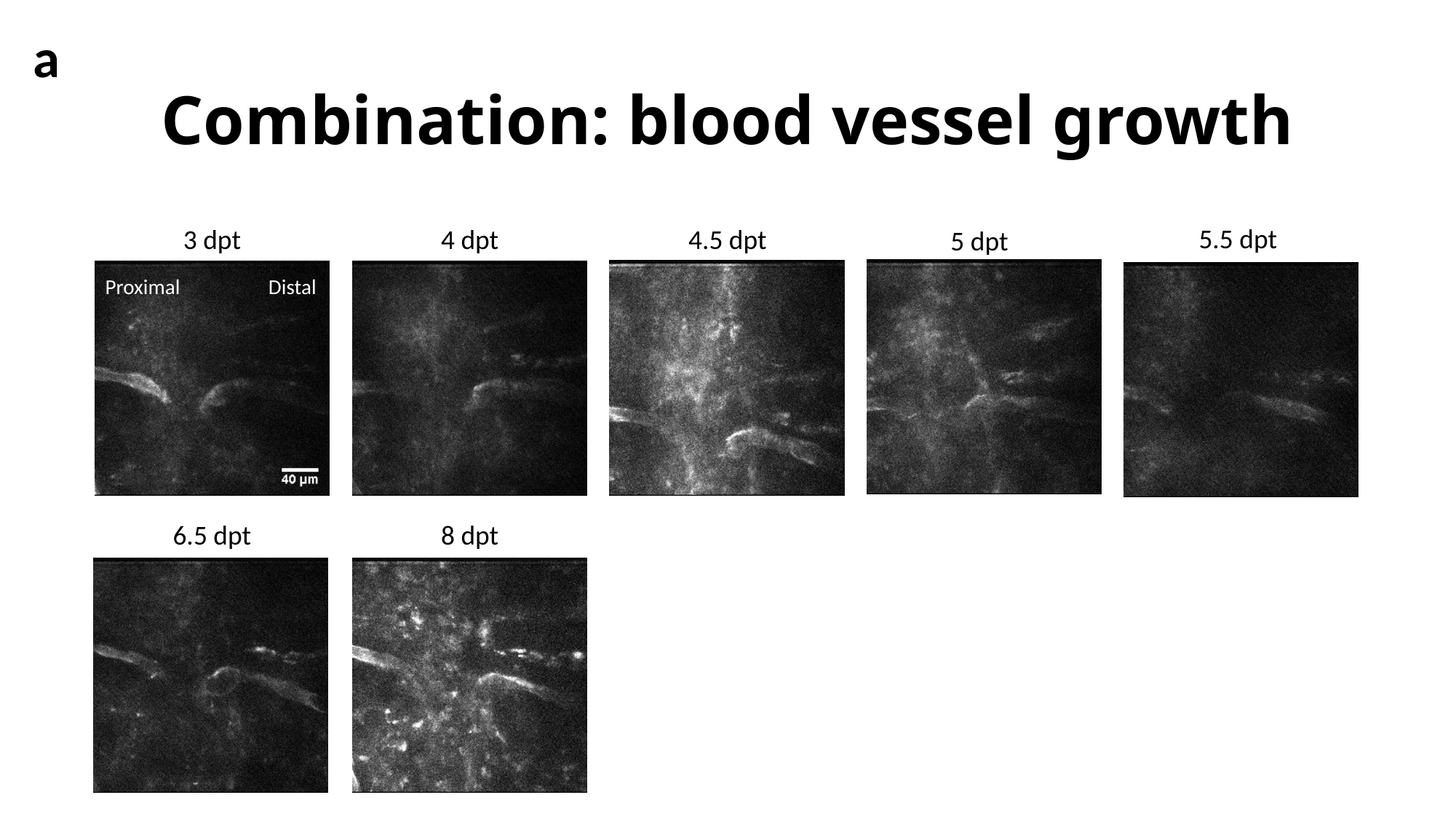

a
# Combination: blood vessel growth
5.5 dpt
4 dpt
4.5 dpt
3 dpt
5 dpt
Distal
Proximal
8 dpt
6.5 dpt

## Slide 3
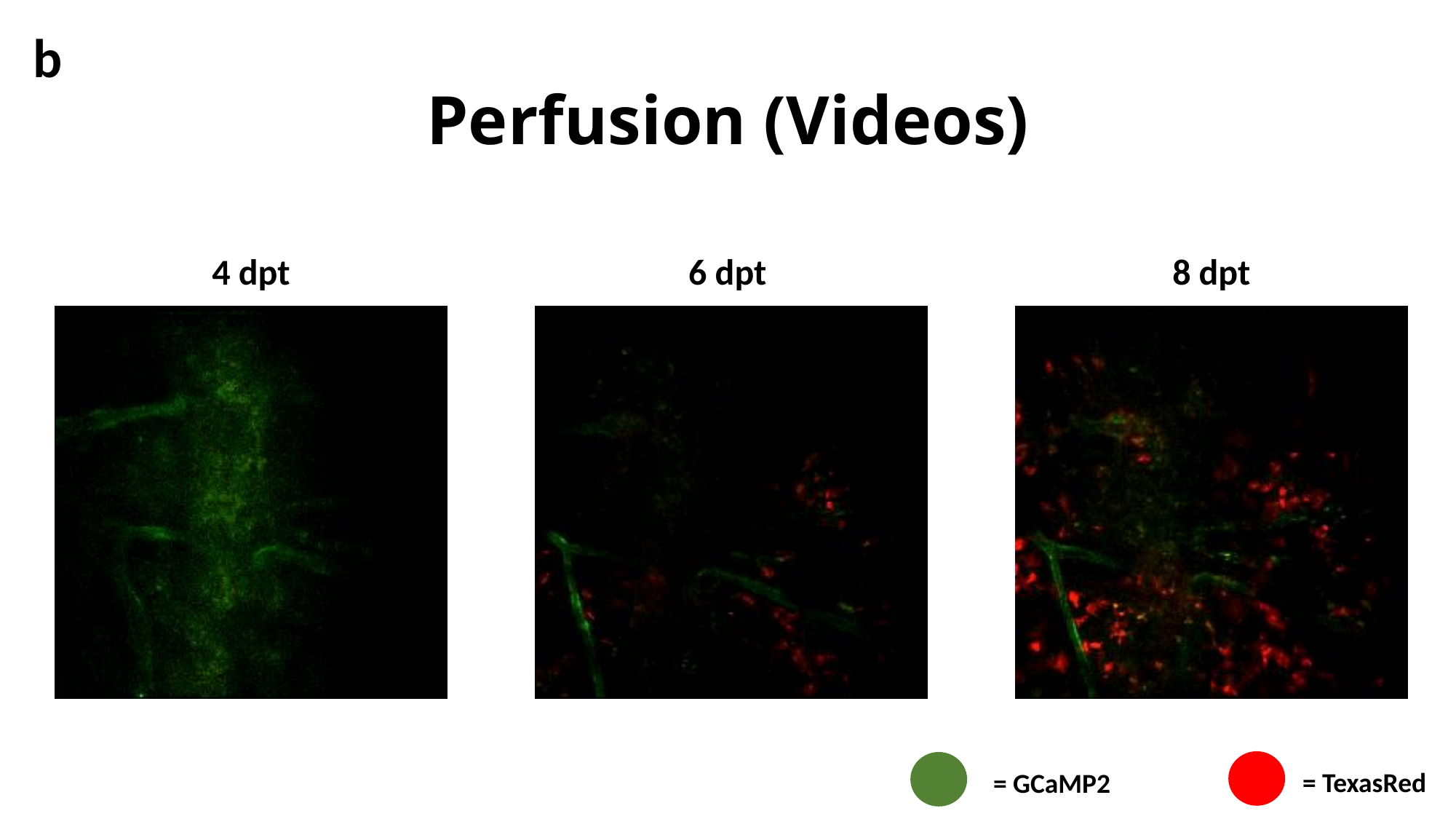

b
# Perfusion (Videos)
4 dpt
6 dpt
8 dpt
= TexasRed
= GCaMP2

## Slide 4
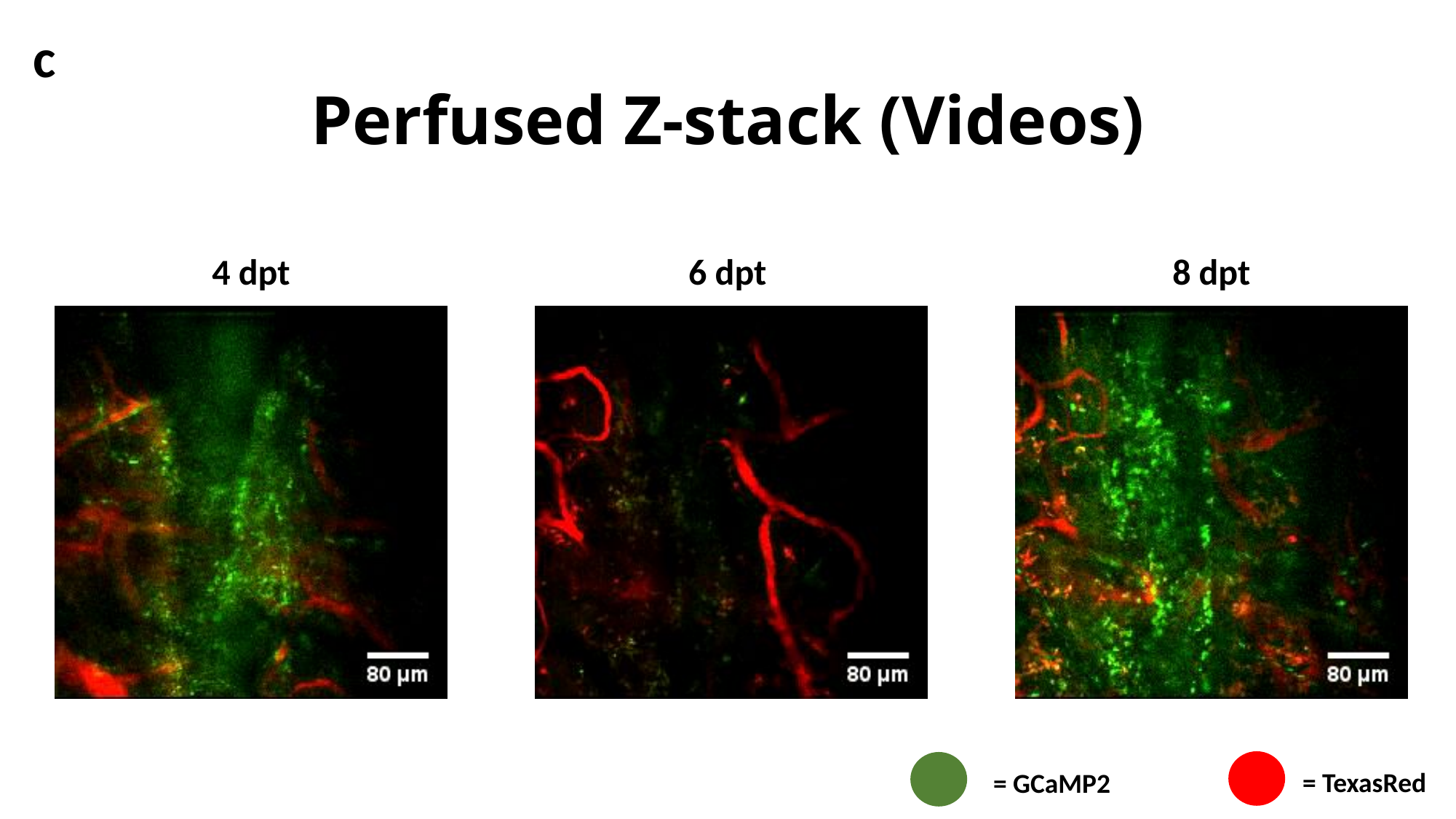

c
# Perfused Z-stack (Videos)
4 dpt
6 dpt
8 dpt
= TexasRed
= GCaMP2
